# Pollution Profiles, Source Identification and Health Risk Assessment of Heavy Metals in Soil near a Non-Ferrous Metal Smelting Plant

**DOI:** 10.3390/ijerph20021004

**Published:** 2023-01-05

**Authors:** Mengdie Qi, Yingjun Wu, Shu Zhang, Guiying Li, Taicheng An

**Affiliations:** 1 Guangdong-Hong Kong-Macao Joint Laboratory for Contaminants Exposure and Health, Guangdong Key Laboratory of Environmental Catalysis and Health Risk Control, Institute of Environmental Health and Pollution Control, Guangdong University of Technology, Guangzhou 510006, China; 2 Guangzhou Key Laboratory of Environmental Catalysis and Pollution Control, Guangdong Technology Research Center for Photocatalytic Technology Integration and Equipment Engineering, School of Environmental Science and Engineering, Guangdong University of Technology, Guangzhou 510006, China

**Keywords:** non-ferrous metal smelting, soil, heavy metals, source identification, risk assessment

## Abstract

Heavy metal pollution related to non-ferrous metal smelting may pose a significant threat to human health. This study analyzed 58 surface soils collected from a representative non-ferrous metal smelting area to screen potentially hazardous heavy metals and evaluate their health risk in the studied area. The findings demonstrated that human activity had contributed to the pollution degrees of Cu, Cd, As, Zn, and Pb in the surrounding area of a non-ferrous metal smelting plant (NMSP). Cu, Cd, As, Zn, Pb, Ni, and Co pollution within the NMSP was serious. Combining the spatial distribution and Spearman correlations with principal component analysis (PCA), the primary sources of Cd, As, Pb, and Zn in surrounding areas were related to non-ferrous metal smelting and transportation activities. High non-cancer (THI = 4.76) and cancer risks (TCR = 2.99 × 10^−4^) were found for adults in the NMSP. Moreover, heavy metals in the surrounding areas posed a potential cancer risk to children (TCR = 3.62 × 10^−6^) and adults (TCR = 1.27 × 10^−5^). The significant contributions of As, Pb, and Cd to health risks requires special attention. The construction of a heavy metal pollution management system will benefit from the current study for the non-ferrous metal smelting industry.

## 1. Introduction

With the rapid advancement of technology, social economy and environmental problems related to industrial pollution have received much attention in recent decades. Heavy metals are the most frequently detected and well-known contaminants. They are toxic, bioaccumulative, and their natural bioremediation is a challenge [1]. The accumulation of metals in an individual’s body can, directly or indirectly, result in many health problems affecting the bones, internal organs, and neurological system [2,3]. The Food and Agriculture Organization of the United Nations/World Health Organization indicates that the long-term intake of inorganic arsenic will lead to cardiovascular disease, type 2 diabetes, and neurotoxicity [4]. Besides being the cause of “itai-itai” disease, chronic cadmium exposure may lead to irreversible renal failure [5,6]. Multiple studies have also discovered that lead adversely influences children’s neurodevelopment [7,8].

Investigations are increasing globally into the pollution of heavy metals in various media, including sediments in rivers or lakes, soil, and air [9,10,11,12,13]. Soil is a primary sink of pollution from wastewater, exhaust gas, and other environmental media [14,15,16]. Compared with other media, soil pollution by heavy metals is more severe.

Among industrial activities, the non-ferrous metal smelting plant (NMSP) is generally considered a critical source of metals, including Cu, Zn, Cd, As, and Pb [17]. Several studies investigated metals’ contamination levels in soils near NMSP. The problem is widely acknowledged internationally in France and Uzbekistan. According to reports, the concentrations of urine arsenic and blood lead in children living close to a smelter in Mexico [18] are higher than the Centers for Disease Control and Prevention action level [19], levels which might cause adverse health effects. Thus, non-ferrous metal smelting activities have resulted in significant heavy metal pollution that may be potentially harmful to people’s health.

Due to the high temperatures involved in the smelting process of non-ferrous metals, some metals may be gasified and suspended in the air [20], eventually settling on the surface soil. The smelting of raw materials and waste residues also disperses metal-containing particles into the atmosphere, with a fate similar to gasified metals [21]. In addition, heavy metals washed out by surface water, such as rainwater, will pollute the soil elsewhere [22], even far from the source. The combination of these disorganized metal emissions and their migration into the environment finally causes exposure to heavy metals to residents living near NMSP. National authorities have been working to control environmental pollution from non-ferrous metal smelting industries. Some plants have been shut down in China and the associated contaminated soil has been remediated. However, as mentioned above, heavy metal remediation is difficult and residual contamination may continue to endanger neighboring residents’ health.

Given this background, this study aimed to investigate the health risks to people living near and within an NMSP. Many reports concern heavy metal pollution in non-ferrous metal smelting sites, but few studies have clarified the division between NMSP and the surrounding areas. This study not only included the health risk assessment of the surrounding residents but also initially revealed that the heavy metal pollution inside an NMSP may have more severe health effects on humans living or working in an NMSP. The main content of this research is as follows: (1) Geoaccumulation indexes (I_geo_) and enrichment factors (EF) were calculated to assess the pollution extent of each sampling site. (2) Kruskal–Wallis H tests were employed to assess the significance of concentration differences between the NMSP, the surrounding areas, and the control areas. (3) Correlation analysis and principal component analysis (PCA) were utilized to clarify the heavy metal sources in soils near the NMSP. (4) The health risks of heavy metals to residents and workers through three contact pathways (inhalation, cutaneous contact, and oral ingestion) were assessed according to the US Environmental Protection Agency (USEPA). The research results will provide a relevant basis for heavy metal contamination control policy in areas related to the NMSP.

## 2. Materials and Methods

### 2.1. Studied Area and Sample Collection

The studied area (114°53′−114°57′ E, 30°9′−30°12′ N) covering 4583 km^2^ has a subtropical monsoon climate with four distinct seasons and abundant rainfall. The prevailing wind is from southeast to northwest, and the annual average wind speed is 2.17 m/s. There are numerous mineral resources in this region. The research area is a typical industrial city that primarily depends on metal smelting and ore mining to support regional economic development. According to local reports, 1,000,000 tons of cathode copper and 700,000 tons of crude copper can still be produced annually in the research area. This study selected the NMSP (114°92′−114°94′ E, 30°17′−30°18′ N) and its surrounding area to investigate the pollution of metals and risks to residents living nearby. In total, 58 topsoil samples were taken in November 2020 from the smelter area (*n* = 10), the adjacent area (*n* = 46), and the control area (*n* = 2) (Figure S1). Five top layers (0–20 cm) of soil were sampled at each sampling site with a stainless-steel shovel. Then, composite soil samples were created by combining these samples. Before analysis, the soils were held in polyethylene plastic bags at −20 °C.

### 2.2. Sample Pretreatment and Metal Analysis

These samples were crushed and sieved using a mesh size of 100 (150 μm) before being lyophilized to eliminate moisture. Samples (0.10 g, accurate to 0.001 g) were combined with 6 mL of 36% (*m*/*m*) hydrochloric acid (ANPEL Laboratory Technologies Inc, Shanghai, China, ppb degree) and 2 mL of 68% (*m*/*m*) nitric acid (Thermo Fisher Scientific, Waltham, MA, USA, ppb degree). Under Microwave Digestion System (MARS6, CEM, Matthews, NC, USA), the sample mixture was treated at 185 °C for 40 min, and the digestive temperature ramp program is listed in Table S1. After cooling, the digested solution was collected and diluted with ultrapure water till it reached 50 mL. Finally, ICP-MS (Agilent 7900, Santa Clara, CA, USA) analysis was performed on 5 mL of the sample liquid after being filtered through the 0.45 μm membrane. The samples must be kept at 4 °C before analysis. This study analyzed nine elements (Cd, Cu, As, Pb, Zn, Ni, Co, V, and Mn). The multiple element standard (160008-01-01) was purchased from o2si, Charleston, SC, USA. The standard released by China’s Ministry of Environmental Protection (MEPC) served as the foundation for this methodology [23].

After each sample treatment, grinding utensils and sieves were rinsed with ultrapure water (resistivity 18.25 MΩ cm) for quality control (QC) and quality assurance (QA). All Teflon microwave digestion vessels and sample containers were immersed in 10% (*v*/*v*) nitric acid overnight and cleaned with ultrapure water (resistivity ≥ 18.25 MΩ cm) at least three times before use. An internal standard containing Sc, Ge, In, and Bi (China Nonferrous Metals and Electronic Materials Analysis and Testing Center) was used to correct the ICP-MS matrix drift. Procedure blanks (2–3 blank samples) were set in each batch, and the average concentration of the blanks was used to adjust the results. Then, 10% of samples were repeatedly analyzed, and a QC sample was inserted every 20 samples. The QC sample was prepared using the national standard (GBW07405, National Standard Detection Research Center, Beijing, China).

The accuracy of the analysis method was further tested using the standard sample, with recovery rates ranging from 81.11% to 94.07% (Figure S2). The method detection limits of Cu, Cd, As, Pb, Ni, Zn, V, Co, and Mn were 3.21, 0.24, 1.14, 0.66, 3.49, 15.80, 1.34, 0.75, and 1.85 mg/kg, respectively.

### 2.3. Data Analysis

#### 2.3.1. Geoaccumulation Index

The contamination extent of heavy metals in soil is measured using the geoaccumulation index (I_geo_). A higher value of I_geo_ indicates more severe contamination in the studied area [14,24,25]. The geoaccumulation index is a tool for evaluating the level of interference from human activity to soil contamination and compensating for environmental background value fluctuations of metal concentrations. I_geo_ is calculated according to Equation (1) [25]:

(1) $$I_{\mathrm{geo}}=\log_{2}\left(\frac{C_{n}}{1.5C_{B}}\right)$$

where C_n_ represents the concentration of metals in soils of the studied region (mg/kg); C_B_ represents the background content of metal n (mg/kg) [26]. To account for potential fluctuations in the background values, the constant 1.5 is utilized [27,28]. Table S2 lists the I_geo_ evaluation standards. The background values of the studied elements are shown in Table 1.

#### 2.3.2. Enrichment Factor

The enrichment factor (EF) evaluates the metal contamination level and is applied to assess the influence degree of anthropological factors on element enrichment in soil [29,30,31]. An EF value greater than two indicates a certain degree of metal enrichment in the studied area. EF is calculated according to Equation (2):

(2) $$\mathrm{EF}=\frac{{\left(C_{n}/C_{\mathrm{ref}}\right)}_{\mathrm{sample}}}{{\left(C_{n}/C_{\mathrm{ref}}\right)}_{\mathrm{background}}}$$

where C_n_ is the content of heavy metals (mg/kg); C_ref_ means the content of reference elements (mg/kg) [26]. Generally, relatively stable, non-volatile, and ubiquitous elements in the crust, such as Sc, are used as reference elements [32]. V was used as the reference element for calculating EF since the concentration in soil samples was stable under the study conditions in the studied area. The evaluation criteria for EF are also listed in Table S2. The background values of the reference element are also listed in Table 1.

#### 2.3.3. Human Health Risk

Risk assessment models were created to estimate the cancer and non-cancer risks of humans. Humans have three main pathways to come into contact with heavy metals in soil (oral, inhalation, and skin absorption). Average daily dose (ADD) formulae for each contact pathway calculate source contribution [33]. The ADD was calculated according to Equations (3)–(7):

(3) $${\mathrm{ADD}}_{s-\mathrm{oral}}=\frac{C_{s}\times {\mathrm{IR}}_{s}\times \mathrm{CF}\times \mathrm{FI}\times \mathrm{EF}\times \mathrm{ED}}{\mathrm{BW}\times \mathrm{AT}}$$

(4) $${\mathrm{ADD}}_{s-\mathrm{inh}}=\frac{C_{s}\times \mathrm{IR}\times \mathrm{EF}\times \mathrm{ED}}{\mathrm{BW}\times \mathrm{AT}\times \mathrm{PEF}}$$

(5) $${\mathrm{ADD}}_{s-\mathrm{dermal}}=\frac{C_{s}\times \mathrm{CF}\times {\mathrm{SA}}_{s}\times \mathrm{AF}\times {\mathrm{ABS}}_{d}\times \mathrm{EF}\times \mathrm{ED}}{\mathrm{BW}\times \mathrm{AT}}$$

(6) $${\mathrm{SA}}_{s}=\mathrm{SAE}\times \mathrm{SER}$$

(7) $${\mathrm{ADD}}_{s}={\mathrm{ADD}}_{s-\mathrm{oral}}+{\mathrm{ADD}}_{s-\mathrm{inh}}+{\mathrm{ADD}}_{s-\mathrm{dermal}}$$

The variables and values utilized in the exposure level assessment are listed in Table S3.

The *Technical Guidelines for Risk Assessment of Contaminated Sites* [34] provide some extrapolation formulae, including for IUR and RfC, which can derive the SF_i_ and RfD_i_ of the respiratory inhalation route. The SF_d_ and RfD_d_ of the skin contact route can be calculated from the SF_o_ and RfD_o_ of the oral ingestion route [34]. The RfD_i_, RfD_d_, SF_i_, and SF_d_ can be calculated according to Equations (8)–(11):

(8) $${\mathrm{RfD}}_{i}=\frac{\mathrm{RfC}\times \mathrm{IR}}{\mathrm{BW}}$$

(9) $${\mathrm{RfD}}_{d}={\mathrm{RfD}}_{o}\times \mathrm{GIABS}$$

(10) $${\mathrm{SF}}_{i}=\frac{\mathrm{IUR}\times \mathrm{BW}}{\mathrm{IR}}$$

(11) $${\mathrm{SF}}_{d}=\frac{{\mathrm{SF}}_{o}}{\mathrm{GIABS}}$$

Table S3 contains the parameters and values used in Equations (8)–(11).

The human health risk evaluation models link heavy metal exposure levels to non-cancer and cancer risk in humans (hazard quotient HQ and CR, respectively) [33]. Total cancer risk (TCR) and total hazard index (THI), which may calculate the overall cancer and non-cancer risks from metals in soils, are the sums of the cancer risk (CR) and hazard index (HI) for various heavy metals. HQ, CR, HI, THI, and TCR are expressed by Equations (12)–(15)

(12) $$\mathrm{HI}=\sum \mathrm{HQ}=\sum \frac{\mathrm{ADD}}{\mathrm{RfD}}$$

(13) THI=∑HI

(14) CR=∑ADD×SF

(15) TCR=∑CR

The parameter values used for non-cancer and cancer risk evaluation are listed in Table S4.

#### 2.3.4. Data Processing and Analysis

Statistical analysis of the data (mean, coefficient of variation (CV), standard deviation (SD), and extreme values) was collated. Kruskal–Wallis H tests of heavy metals between the different areas and Spearman correlation analysis between heavy metals were performed using SPSS 22. High correlation of heavy metals might demonstrate that these elements’ sources are similar [35]. Inverse distance interpolation was employed to assess the spatial distribution of heavy metals based on the concentration, latitude, and longitude of sampling points using geographic information system (GIS) software (ArcGIS 10.2). The PCA method conducted by SPSS was applied to analyze more accurately the origin of metals in the soil [36].

## 3. Results and Discussion

### 3.1. Pollution Characteristics of Heavy Metals in Soils of the NMSP and Surrounding Area

Table 1 and Table S5 summarize the descriptive statistical findings for the presence of heavy metals (Cu, Cd, As, Zn, Pb, Ni, Co, V, and Mn) in soils from NMSP, surrounding area, and the control area. The surrounding area’s contents of Cu, Zn, As, Cd, and Pb were higher than the control area’s and the investigated area’s background levels, with median contents of 264.96, 196.30, 28.44, 2.06, and 74.62 mg/kg, respectively. Notably, the median concentrations of Cu and Cd were approximately 5.3 and 5.1 times higher than the risk screening values of soils for agriculture (Table S6). Moreover, Zn, As, and Pb in 47.83%, 47.83%, and 45.65% of samples were greater than the risk screening values. These results suggest pollution of Zn, Cu, As, Cd, and Pb in the surrounding area.

The pollution of Zn, Cu, As, Pb, and Cd in this study was compared with that of heavy metals in soils related to the non-ferrous metal industry in other countries (Table S7). Ni, Cu, Zn, and Cd concentrations in this research were greater than those in the soil of Legnica (southwestern Poland), while the contents of Pb were comparable [37]. Compared with heavy metal contamination in a region of Bulgaria nearby the largest copper smelter in Srednogorie [38], Cd content in this study’s surrounding area is higher. The comparison results indicate that metals contaminate the surrounding area in this study more than in other countries.

**Figure 1:**
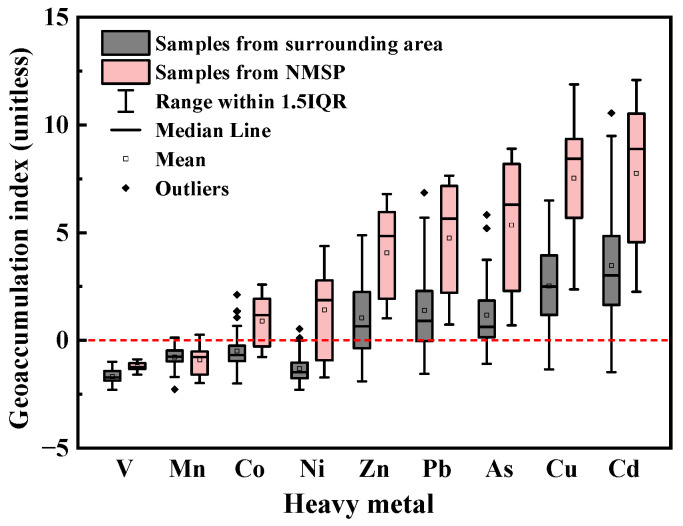
Geoaccumulation index (I_geo_) values for heavy metals in soils of surrounding area and non-ferrous metal smelting plant (NMSP).

**Figure 2:**
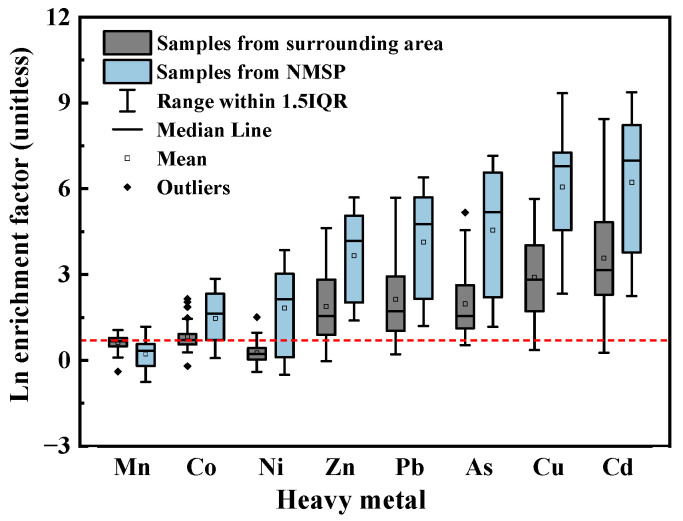
Box-plots of the natural logarithm of the enrichment factor (EF) values for heavy metals in soils of surrounding area and non-ferrous metal smelting plant (NMSP).

According to Figure 1, the surrounding area’s median I_geo_ levels for heavy metals were as follows: Cd (3.02) > Cu (2.50) > Pb (0.90) > As (0.62) > Zn (0.65) > 0. The median EF values of these five metals follow a similar order (Figure 2): Cd (23.47) > Cu (16.86) > Pb (5.59) > Zn (4.71) > As (4.68) > 2. The I_geo_ results demonstrate that surrounding area soils were heavily contaminated by Cd, moderately to heavily contaminated with Cu, and uncontaminated to moderately contaminated by Zn, Pb, and As. Furthermore, with EF values larger than 2, contamination of Zn, Cu, Cd, As, and Pb in surrounding regions was caused by human activities. Moreover, Table 1 shows the CVs for Zn, Cu, Cd, As, and Pb in surrounding areas, which were all relatively high (CV > 1.00), demonstrating that anthropogenic activities have a considerable effect on concentrations of metals in soils of surrounding regions [39]. To sum up, the surrounding regions’ soil was contaminated by Zn, Cu, As, Pb, and Cd with a possible correlation to human activities.

There are various sources of heavy metals in the surrounding area. An NMSP is often considered a primary source of soil contamination with heavy metals [40]. Heavy metals discharged from non-ferrous metal smelting can pollute the surrounding soil through atmospheric deposition, rainfall flushing, and weathering [41,42,43]. Table 1 shows that median contents of Co, Ni, Cu, Zn, As, Cd, and Pb in soils from NMSP were 53.88, 222.24, 16,044.90, 4247.79, 1910.62, 154.14, and 2097.49 mg/kg, respectively. Samples from the NMSP notably have more of these seven metals than those from surrounding locations (Kruskal–Wallis H tests, *p* < 0.05). The median contents of As, Cd, and Pb in the NMSP were 31.8, 2.4, and 2.6 times higher than the risk screening data for soil contamination of industrial land (Table S6). Additionally, the risk screening levels were exceeded by the contents of Cu, Co, and Ni in 50.00%, 30.00%, and 20.00% of the samples taken from the NMSP. The median values of I_geo_ and EFs for the NMSP’s soil followed similar orders in surrounding areas. The median I_geo_ values of investigated elements (Figure 1) followed a descending order: Cd (8.89) > Cu (8.43) > As (6.30) > Pb (5.64) > Zn (4.84) > Ni (1.86) > Co (1.16) > 0. The median EF values of Cd (1300.70), Cu (905.18), As (222.25), Pb (120.09), Zn (73.48), Ni (9.03), and Co (5.21) in NMSP are shown in Figure 2. These results indicate that the soil in NMSP was extremely contaminated by Cd, Cu, As, and Pb, heavily to extremely contaminated by Zn, and contaminated by Co and Ni. Human activities might be why there are high soil enrichments of Cu, Cd, Pb, As, Zn, Co, and Ni in the NMSP.

Notably, similar to other studies on NMSP, the contamination of Cd in this study is more severe than that of Cu [44,45]. The high enrichment of Cd may be because, although the main product of non-ferrous metal smelting is copper, the overall process requires a high Cu recovery rate, resulting in less environmental Cu pollution. Meanwhile, Cd in soil media has high mobility and release potential [46]. Therefore, since the whole smelting process does not focus on the recovery of Cd, this metal may be enriched in NMSP soils. Moreover, Cd’s properties could account for higher pollution than Cu in the surrounding area. Overall, soil Cd pollution related to non-ferrous metal smelting deserves more attention.

**Figure 3:**
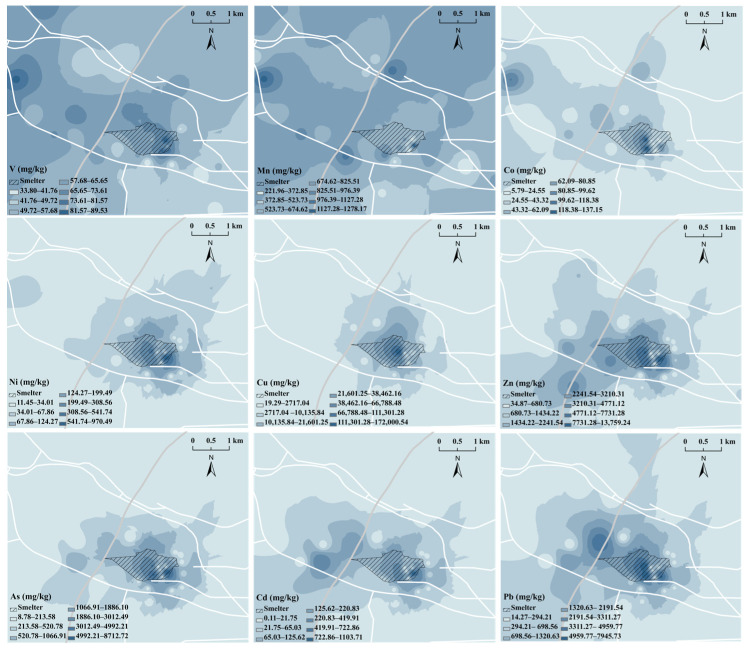
Spatial distribution of heavy metals in soils of the surrounding area and non-ferrous metal smelting plant (NMSP).

To learn more about the heavy metal contamination features and likely sources in the NMSP and surrounding area, spatial distribution maps are illustrated in Figure 3. As shown, the concentrations of Zn, Cu, Cd, As, and Pb reduced with the increase in distance from the NMSP into the surrounding area. Meanwhile, Zn, As, Cd, and Pb pollution primarily accumulated in the western region of the NMSP, consistent with the prevailing wind direction of the studied areas. Additionally, previous research has discovered a strong link between the distribution of metals and the predominant wind direction in the investigated regions [47]. The migration and sedimentation of airborne particles due to industrial emissions and other pollution sources may cause this [48,49]. These results indicate that the NMSP is a point source of these pollutions in surrounding areas. The spatial distribution of Cu in the surrounding regions was relatively narrow, possibly caused by the high recovery of Cu in smelting processes. The reason for Cu’s high enrichment in NMSP needs to be further analyzed.

The values of the metals under investigation in two samples from the control sites are displayed in Table S5. Those were below the agricultural land risk screening levels (Table S6). Compared to the control areas, the surrounding area’s mean metal concentrations were greater, showing that anthropogenic activities impacted metals in the soils of surrounding regions. In particular, the contents of Cd, Cu, Zn, As, and Pb were 88.6, 14.5, 9.8, 7.6, and 5.7 times higher in the surrounding zone than in the control region, respectively.

### 3.2. Source Analysis of Heavy Metals in Soils of the NMSP and Surrounding Areas

PCA combined with Spearman correlation were used to clarify the origins of these heavy metals in the NMSP and surrounding regions. After varimax rotation, the two principal components (PC) for heavy metals in the soil from the NMSP and surrounding area with eigenvalues higher than one were extracted.

**Figure 4:**
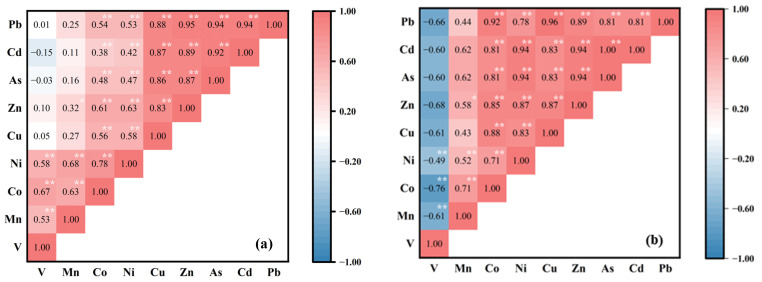
Spearman correlation hot map between heavy metals in soils of (**a**) surrounding area and (**b**) non-ferrous metal smelting plant (NMSP). *: probability value < 0.05; **: probability value < 0.01.

Heavy metals in soils of the surrounding areas were divided into two components, explaining 73.30% of the total variance (Table 2). Zn (0.75), As (0.97), Cd (0.91), and Pb (0.86) made up the first principal component (PC1), explaining 38.86% of the total variance. Meanwhile, the correlation between Pb, Cd, As, and Zn was significantly positive (*p* < 0.01 and r > 0.80) with each other (Figure 4a), suggesting that Cd, Pb, As, and Zn might have a similar source. Meanwhile, as shown in spatial distribution, the NMSP was an obvious point source of these four heavy metals (Figure 3). The literature confirms that the soil contamination by Pb, As, Cd, and Zn as well as industrial smelting emissions are inextricably linked [46,50,51]. Moreover, in contrast to the study areas where non-industrial factors such as transportation are the primary sources of heavy metal [52,53], the contamination levels of As and Cd, which are characteristic pollutants of non-ferrous metals, were more serious in surrounding areas in this study. The severe pollution of As and Cd also indicates that this study’s industrial point source NMSP greatly influences heavy metal content in the soil from surrounding areas. However, due to the current busy transportation in the studied area [45], it cannot be excluded that gasoline combustion and automobile tire wear may be potential contributors to Zn and Pb in the soil [54]. Therefore, PC1 might be related to non-ferrous metal smelting and transportation activities.

The PC2, accounting for 34.44% of the overall variation, showed positive loading for V (0.76), Mn (0.87), Co (0.79), Ni (0.79), and Cu (0.66). Moreover, the correlation between V, Mn, Co, and Ni was relatively weak (r < 0.80) but still significant (*p* < 0.01) (Figure 4a), demonstrating that the presence of these metals in the soil of the surrounding region has similar origins. As shown in the spatial distribution, V and Mn were relatively dispersed and their concentrations had no apparent relationship to the distance from the NMSP (Figure 3). The result indicates that, in the studied area, no clear correlation exists between V and Mn and industrial smelting activities. Moreover, the distributions of Co and Ni in the surrounding areas were not similar to other metals related to smelting activities, such as Zn and As. Combined with the pollution characteristics in the surrounding area, there was no or only slight pollution of V, Mn, Co, and Ni in the studied soils. Therefore, PC2 for the surrounding areas could come from natural sources.

In NMSP, the CVs for Cu, As, Cd, Pb, Ni, Zn, and Co were high (Table 1), meaning that anthropogenic emissions may have a substantial effect on metal content in soil [55]. The human activities which cause high enrichment in the NMSP soil might be related to the non-ferrous metal smelting activities. In contrast, V was negatively correlated (*p* < 0.05) with Zn (r = −0.68) and Co (r = −0.76). This result corroborates the above discussion about the natural source of V in the NMSP.

The PCA explained 86.73% of the total variance for soil samples in the NMSP (Table 2). Interestingly, V has a negative loading on both components, which can indirectly indicate that the components extracted by PCA in the NMSP soil may be affected by different human factors. Specifically, PC1 was primarily related to Mn, Ni, Zn, As, and Cd, which gave values of 0.83, 0.93, 0.82, 0.93, and 0.97. Meanwhile, Ni, Zn, As, and Cd were significantly positively correlated (*p* < 0.01 and r > 0.8) with each other (Figure 4b), suggesting that the As, Zn, and Cd in NMSP soils were probably primarily from the same source. As discussed, smelting could release Ni, Zn, As, and Cd into the air as particulate dust formed during ore processing and the volatilization of semi-raw metals caused by high temperatures [56]. The spherical particles condensed during the high-temperature smelting process can deposit onto the soil surface from the atmosphere, and metals are released after weathering [57]. As a result, large emissions could cause Ni, Zn, As, and Cd pollution in this NMSP. PC2 for the NMSP explained 38.23% of the total variance, demonstrating a loading of Co, Cu, and Pb. Moreover, significantly positive correlations between Co, Cu, and Pb were observed (*p* < 0.01) (Figure 4b). As mentioned above, the main product of this NMSP is copper. However, smelted copper tailing also contains lead and cobalt [44,58]. Therefore, PC2 might be derived from the long-term disorganized stacking of smelting raw materials or tailings, resulting in severe pollution of Co, Cu, and Pb in the NMSP soils.

The PCA analysis indicated that smelting-related activities cause Cd, Cu, As, Pb, Zn, Ni, and Co pollution in NMSP. This conclusion confirms the previous inference that Cd, Pb, As, and Zn in surrounding regions might also be derived from non-ferrous metal smelting-related activities. Therefore, smelting activities are inseparable from the emission and enrichment of As, Cd, Zn, and Pb in NMSP as well as the surrounding areas.

### 3.3. Human Health Risks from the NMSP and Surrounding Area

Considering the pollution levels and risks to residents’ health in a studied area is critical. Seven heavy metals were chosen to perform a health risk assessment for the NMSP and surrounding area: Cu, Cd, Pb, Zn, As, Ni, and Co. The ADD values for cancer and non-cancer risks (Tables S8 and S9) demonstrated that the main exposure pathway of heavy metal is oral ingestion, consistent with previous studies [59]. Meanwhile, a comparison of ADD values for adults and children indicated that children have greater total exposure levels than adults. The results demonstrate that children have a higher probability of contacting metals in soil, perhaps owing to lower proximity to the ground and hand-matching activity [60].

**Figure 5:**
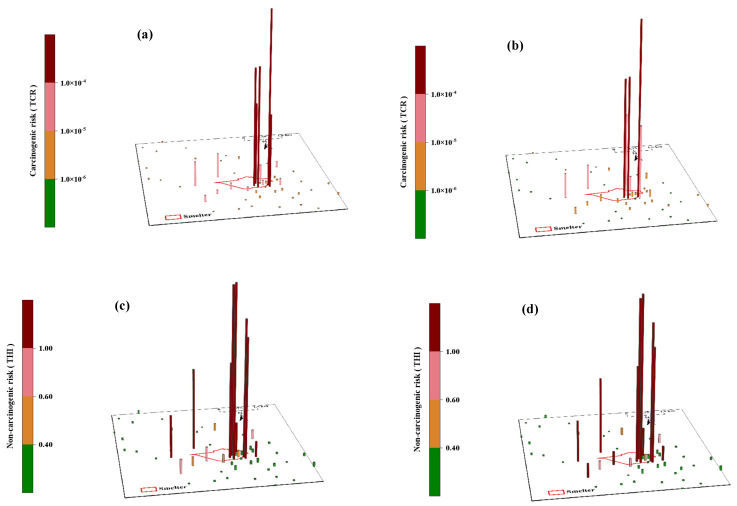
Health risk assessment for sampling sites of surrounding areas and non-ferrous metal smelting plant (NMSP): (**a**) cancer risk for adults, (**b**) cancer risk for children, (**c**) non-cancer risk for adults, and (**d**) non-cancer risk for children.

In the surrounding areas, heavy metals in soil have cancer and non-cancer risks that cannot be underestimated. As shown in Figure 5, about 2−4 sampling spots presented non-cancer risks to people in the surrounding areas. The result agreed with the spatial distribution of metals in this study. Additionally, adults and children had a mean TCR of 1.27 × 10^−5^ and 3.62 × 10^−6^, respectively (Figure S3), meaning that heavy metals in the surrounding areas still have potential cancer risks for the residents. Fortunately, the mean THI in the surrounding regions was below the safe limit (THI = 1.00) [61], proving that humans suffered no non-cancer risks in the surrounding areas.

In the studied area, NMSP was where the majority of the examined area’s cancer and non-cancer risks were discovered (Figure 5), with THI and TCR values greater than the safe limit (THI = 1.00, TCR = 1.00 × 10^−4^) [61]. About 60% of the sampling spots in the NMSP had non-cancer and cancer risks (Figure 5). Children and adults had mean TCR values of 8.50 × 10^−5^ and 2.99 × 10^−4^, respectively (Figure S3), indicating that the heavy metals in NMSP pose cancer risk for adults and that the cancer risk for kids was potential but not negligible. The cancer risk (CR) for individual elements shows that the contribution of As was 86.72%, Cd was 31.63%, and Pb was 0.42%. Moreover, the non-cancer risks in NMSP also deserve significant attention, with non-cancer risks for both adults and children (THI = 4.76 and 5.68, respectively) (Figure S3). Individually, adults and children may have non-cancer risk from As and Pb in the NMSP soil, where the mean HI values are 1.4–3.6 times higher than the safe limit (Figure S3).

Overall, the surrounding area poses potential health risks from heavy metals. Furthermore, particular focus should be placed on the NMSP’s non-cancer and cancer risks, which are much higher than the safe limits. Strategies to control heavy metals pollution in the NMSP and its surrounding areas should focus on As, Cd, and Pb. As NMSP is the pillar industry for the regional economy, it is necessary to further control and repair the metal pollution of non-ferrous smelting to the surrounding soil environment.

## 4. Conclusions

This study describes the contamination features of metals and potential health risks in an NMSP and its surrounding areas. Estimated I_geo_ and EF values revealed that Cd heavily contaminated soil from the surrounding regions, and Zn, Cu, As, and Pb heavily to moderately contaminated it, enrichments possibly caused by human activities. Moreover, severe contamination by Cd, Cu, As, Pb, Zn, Ni, and Co is present within the NMSP. By combining the PCA results of the NMSP and surrounding area, this study finds that the pollution of the surrounding area might mainly originate from non-ferrous metal smelting-related industrial activities and transportation. Furthermore, the potential source of the heavy metals in NMSP was likely the long-term disorganized raw materials and tailings stacking. Lastly, the soil’s heavy metals in this research pose non-cancer and cancer risks to people, especially to the workers in the NMSP. As, Cd, and Pb ought to be listed as metals for priority control in this region due to their high contribution to non-cancer and cancer risks. The paper’s conclusion can be utilized as a reference for heavy metal target control in the non-ferrous metal smelting industrial zone. The correct storage of raw ore and tailings, as well as the reduction of emissions and enhancement of emission treatment during the smelting process, are crucial for the non-ferrous smelting industries.

## Figures and Tables

**Table 1:** Descriptive statistics of heavy metals in soils of the surrounding area and non-ferrous metal smelting plant (NMSP).

Element	Min	Median	Max	Mean	Standard Deviation	Coefficient of Variation	BGV
Surrounding area							
V	33.78	50.32	83.16	52.68	10.42	0.20	110.20
Mn	221.19	634.84	1159.72	647.07	180.10	0.28	712.00
Co	5.78	14.38	100.18	19.79	16.66	0.84	15.40
Ni	11.43	20.02	81.12	25.18	13.76	0.55	37.30
Cu	18.18	264.96	4160.19	583.37	820.66	1.41	30.70
Zn	33.64	196.30	3676.61	606.95	906.35	1.49	83.60
As	8.65	28.44	1049.96	92.30	181.05	1.96	12.30
Cd	0.09	2.06	382.28	19.38	61.16	3.16	0.17
Pb	13.61	74.62	4642.77	303.55	732.05	2.41	26.70
NMSP							
V	54.88	70.32	89.59	71.70	10.02	0.14	110.20
Mn	270.67	625.15	1283.20	626.72	276.11	0.44	712.00
Co	13.58	53.88	138.89	60.37	45.07	0.75	15.40
Ni	16.94	222.24	1163.26	343.60	387.25	1.13	37.30
Cu	238.24	16,044.90	173,887.37	31,627.73	49,699.53	1.57	30.70
Zn	254.13	4247.79	13,827.58	4789.46	4495.54	0.94	83.60
As	29.87	1910.62	8759.41	2763.95	2943.77	1.07	12.30
Cd	1.22	154.14	1110.12	316.33	402.19	1.27	0.17
Pb	66.53	2097.49	8017.90	3026.90	3004.71	0.99	26.70

BGV: background value of soil heavy metal concentration in the studied area [26].

**Table 2:** Component matrix for heavy metals in soils of the surrounding area and non-ferrous metal smelting plant (NMSP).

Metal	Surrounding Area		NMSP	
	PC1	PC2	PC1	PC2
V	−0.09	0.76	−0.27	−0.74
Mn	−0.03	0.87	0.83	0.25
Co	0.34	0.79	0.42	0.87
Ni	0.37	0.79	0.93	0.11
Cu	0.41	0.66	−0.02	0.92
Zn	0.75	0.15	0.82	0.53
As	0.97	0.10	0.93	0.34
Cd	0.91	0.08	0.97	0.12
Pb	0.86	0.19	0.30	0.89
Eigenvalue > 1	3.50	3.10	4.36	3.44
% of variance	38.86	34.44	48.50	38.23
Cumulative%	38.86	73.30	48.50	86.73

Extraction method: principal component analysis.

## Data Availability

Not applicable.

## Supplementary Materials

The following supporting information can be downloaded at: https://www.mdpi.com/article/10.3390/ijerph20021004/s1, Figure S1. Map of the studied area and location of soil sampling sites (*n* = 56); Figure S2. The recovery rates of heavy metal in the standard sample; Figure S3. Health risk assessment for soil of surrounding area and non-ferrous metal smelting plant (NMSP): (a) cancer risk and (b) non-cancer risk; Table S1. Microwave digestion reference procedure; Table S2. Classification of geoaccumulation index (I_geo_) and Enrichment factor (EF); Table S3. Parameter meaning and selected value of the average daily intake of heavy metals in soil; Table S4. Parameters for non-cancer risk and cancer risk assessment; Table S5. The concentrations of heavy metals in the control area (mg/kg); Table S6. Summary of the risk screening standards levels (mg/kg) for heavy metals under investigation; Table S7. The median concentrations of heavy metals in investigations of other countries; Table S8. The exposure level of the adults and children in the surrounding area and non-ferrous metal smelting plant (NMSP) for non-cancer risks assessment; Table S9. The exposure level of the adults and children in the surrounding area and non-ferrous metal smelting plant (NMSP) for cancer risks assessment. References [25,34,37,38,45,62,63,64,65,66,67,68,69] are cited in the Supplementary Materials.
