# Supplementary material for: Pollution Profiles, Source Identification and Health Risk Assessment of Heavy Metals in Soil near a Non-Ferrous Metal Smelting Plant
Source: Int J Environ Res Public Health. 2023 Jan 5;20(2):1004. doi: 10.3390/ijerph20021004 (PMC9858899; doi:10.3390/ijerph20021004)
Supplement: Supplementary file 1 [file ijerph-20-01004-s001.zip › ijerph-2030087-supplementary.pdf]

Supporting information (SI):

Pollution Profiles, Source Identification and Health Risk Assessment of Heavy Metals in Soil Near a Non-Ferrous Metal Smelting Plant

Mengdie Qi[1,2], Yingjun Wu[1,2], Shu Zhang[1,2], Guiying Li[1,2,*], Taicheng An[1,2]

[1] *Guangdong-Hong Kong-Macao Joint Laboratory for Contaminants Exposure and Health, Guangdong Key Laboratory of Environmental Catalysis and Health Risk Control, Institute of Environmental Health and Pollution Control, Guangdong University of Technology, Guangzhou, 510006, China;*

[2] *Guangzhou Key Laboratory of Environmental Catalysis and Pollution Control, Guangdong Technology Research Center for Photocatalytic Technology Integration and Equipment Engineering, School of Environmental Science and Engineering, Guangdong University of Technology, Guangzhou, 510006, China.*

* Corresponding author: **Prof. Guiying Li**, *E-mail:* ligy1999@gdut.edu.cn

Figures = 3

Tables = 9

List

1. Figure S1 Map of the studied area and location of soil sampling sites (n = 56).
2. Figure S2 The recovery rates of heavy metal in the standard sample.
3. Figure S3 Health risk assessment for soil of surrounding area and non-ferrous metal smelting plant (NMSP): (a) cancer risk and (b) non-cancer risk.
4. Table S1 Microwave digestion reference procedure.
5. Table S2 Classification of geoaccumulation index (I_{geo}) and Enrichment factor (EF).
6. Table S3 Parameter meaning and selected value of the average daily intake of heavy metals in soil.
7. Table S4 Parameters for non-cancer risk and cancer risk assessment.
8. Table S5 The concentrations of heavy metals in the control area (mg/kg).
9. Table S6 Summary of the risk screening standards levels (mg/kg) for heavy metals under investigation.
10. Table S7 The median concentrations of heavy metals in investigations of other countries.
11. Table S8 The exposure level of the adults and children in the surrounding area and non-ferrous metal smelting plant (NMSP) for non-cancer risk assessment.
12. Table S9 The exposure level of the adults and children in the surrounding area and non-ferrous metal smelting plant (NMSP) for cancer risk assessment.

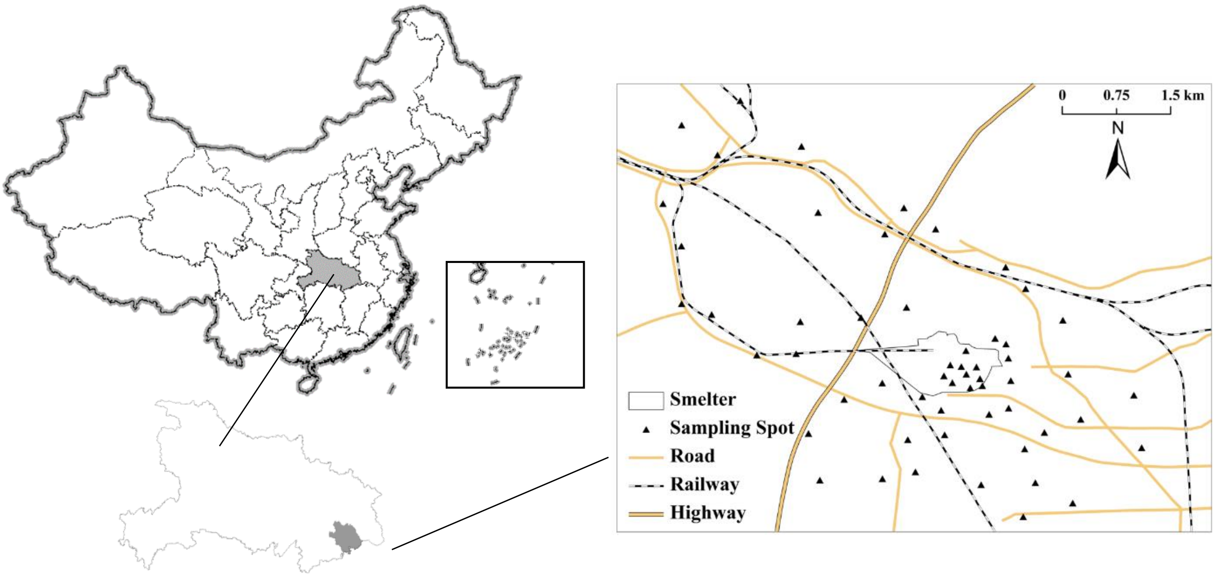

Figure S1. Map of the studied area and location of soil sampling sites (n = 56).

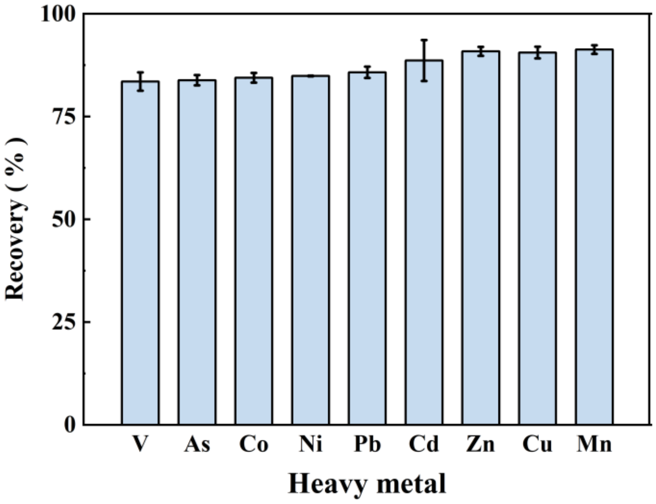

Figure S2. The recovery rates of heavy metal in the standard sample.

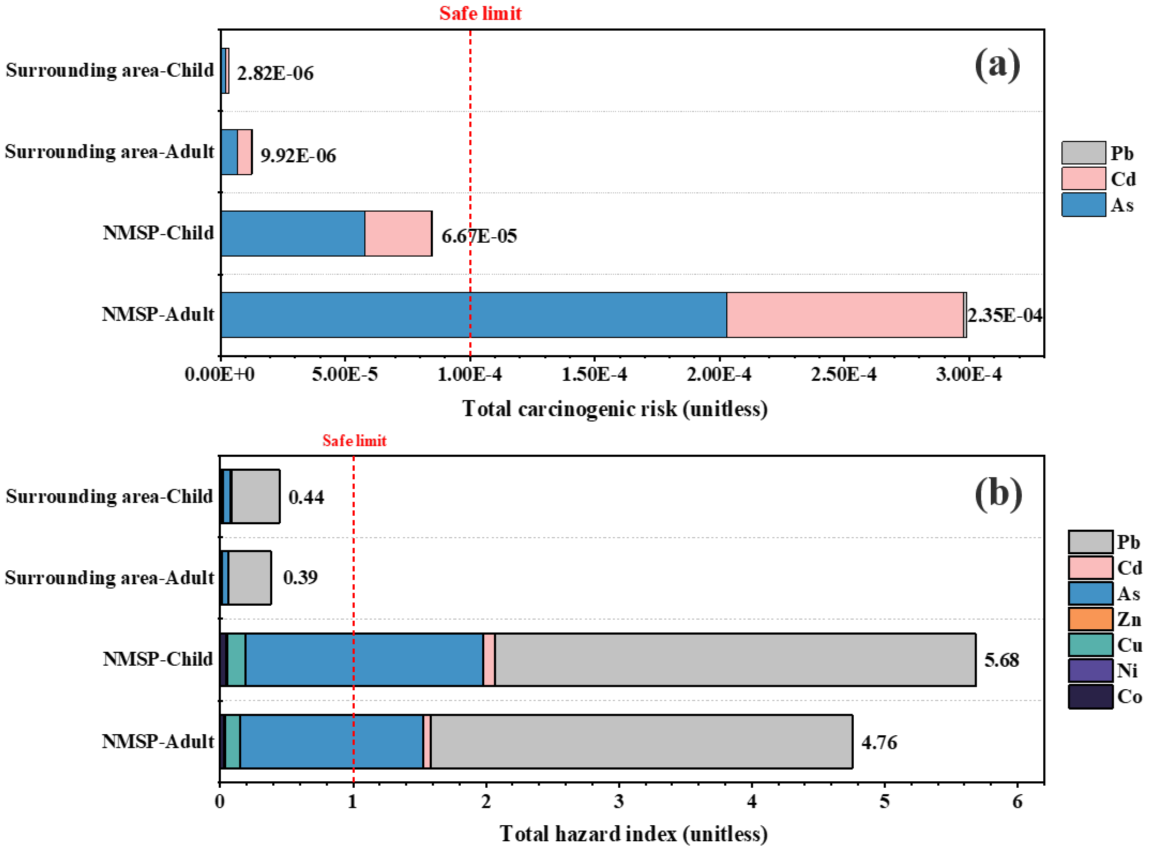

Figure S3. Health risk assessment for soil of surrounding area and non-ferrous metal smelting plant (NMSP): (a) cancer risk and (b) non-cancer risk.

Table S1 Microwave digestion reference procedure [1].

Step	Heating-up time (min)	Target temperature (℃)	Retention time (min)
1	5	120	2
2	4	150	5
3	5	185	40

Table S2 Classification of geoaccumulation index (I_{geo}) and Enrichment factor (EF).

Index	Value	Degree	Reference
Geoaccumulation index	$I_{geo} \leq 0$	uncontaminated	[2]
	$0 < I_{geo} \leq 1$	uncontaminated to moderately contaminated	
	$1 < I_{geo} \leq 2$	moderately contaminated	
	$2 < I_{geo} \leq 3$	moderately to heavily contaminated	
	$3 < I_{geo} \leq 4$	heavily contaminated	
	$4 < I_{geo} \leq 5$	heavily to extremely contaminated	
	$I_{geo} > 5$	extremely contaminated	
Enrichment factor	EF < 2	minimal enrichment	[3]
	2 ≤ EF < 5	moderate enrichment	
	5 ≤ EF < 20	significant enrichment	
	20 ≤ EF < 40	very high enrichment	
	EF ≥ 40	extremely high enrichment	

Table S3 Parameter meaning and selected value of the average daily intake of heavy metals in soil.

Parameters	Parameter meaning	Unit	Children	Adult
ED	Exposure duration	year	6 [a]	24 [a]
BW	Body weight	kg	26.3 [b]	60.1 [c]
IR	Daily inhalation rate	m^3/day	10.2	15.8
SAE	Skin surface area	cm^2	9800	16000
SER	Skin exposure ration	-	0.36	0.32
Cs	Concentrations of potential toxic elements	mg/kg		
IRs	ingestion rate of soil	mg/d	50 [a]	
CF	Conversion factor of concentrations	kg/mg	1.00E-06 [a]	
FI	Oral absorption of soil absorption factors	-	1 [a]	
EF	Exposure frequency	day/year	350 [a]	
PEF	Particulate emission factor	m^3/kg	1.36×10^9 [a]	
AF	Dermal adherence factor	-	0.2 [a]	
ABS_d	Dermal absorption factor	-	As (0.03) [a]<br>Others (0.001) [a]	
RfDi	The reference dose	mg/kg/d		
RfC	Respiratory inhalation reference concentration	mg/m^3		
GIABS	Digestive tract absorption efficiency factor	-	Table S3	
SF	Carcinogenic slope factor	mg/kg/d		
IUR	Respiratory inhalation unit carcinogen	m^3/kg		

[a] [4], [b] [5], [c] [6], [d] [7]

Table S4 Parameters for non-cancer risk and cancer risk assessment.

Elements	IUR ($\mu g/m^3$)$^{-1}$	RfD_o (mg/kg-day)	RfC (mg/kg-day)	RfD_i (mg/m^3)		RfD_d (mg/kg-day)	GIABS	SF_o (mg/kg-day)$^{-1}$	SF_i		SF_d
				adult	child				adult	child	
V	8.30E-03 [a]	9.00E-03 [a]	7.00E-06 [a]	1.69E-06	2.00E-06	2.34E-04	2.60E-02 [a]	n/a	34.40	29.11	n/a
Mn	n/a	1.40E-01 [a]	5.00E-05 [a]	1.21E-05	1.43E-05	1.40E-01	1.00E+00 [a]	n/a	n/a	n/a	n/a
Co	9.00E-03 [a]	3.00E-04 [a]	6.00E-06 [a]	1.45E-06	1.71E-06	3.00E-04	1.00E+00 [a]	n/a	37.30	31.56	n/a
Ni	2.60E-03 [a]	2.00E-02 [a]	9.00E-05 [a]	2.17E-05	2.57E-05	8.00E-04	4.00E-02 [a]	n/a	10.78	9.12	n/a
Cu	n/a	4.00E-02 [a]	n/a	n/a	n/a	4.00E-02	1.00E+00 [a]	n/a	n/a	n/a	n/a
Zn	n/a	3.00E-01 [a]	n/a	n/a	n/a	3.00E-01	1.00E+00 [a]	n/a	n/a	n/a	n/a
As	4.30E-03 [a]	3.00E-04 [a]	1.50E-05 [a]	3.62E-06	4.28E-06	3.00E-04	1.00E+00 [a]	1.50E+00 [a]	17.82	15.08	1.50E+00
Cd	1.80E-03 [a]	1.00E-04 [a]	1.00E-05 [a]	2.41E-06	2.85E-06	2.50E-06	2.50E-02 [a]	6.10E+00 [b]	7.46	6.31	2.44E+02
Pb	1.20E-05 [a]	3.50E-03 [b]	n/a	n/a	n/a	3.50E-03	1.00E+00 [a]	8.50E-03 [a]	0.05	0.04	8.50E-03

n/a, data not available. [a] [8]; [b] [9].

Table S5 The concentrations of heavy metals in the control area (mg/kg).

Sample	V	Mn	Co	Ni	Cu	Zn	As	Cd	Pb
1	23.78	114.30	2.06	7.13	6.44	53.74	12.19	n/a	79.06
2	51.41	763.49	14.20	13.67	72.18	67.76	11.51	0.21	26.09
Mean	37.60	438.90	8.13	10.40	39.31	60.75	11.85	0.21	52.58

n/a, data was below the LOD

Table S6 Summary of the risk screening standards levels (mg/kg) for heavy metals under investigation.

Category	V	Mn	Co	Ni	Cu	Zn	As	Cd	Pb
Chinese standard for agricultural soil[a]	n/a	n/a	n/a	70.00	50.00	200.00	30.00	0.40	100.00
Chinese standard for industrial soil[b]	165.00	n/a	70.00	900.00	18000.00	n/a	60.00	65.00	800.00

n/a, data not available. . [a] [10] [b] [11]

Table S7 The median concentrations of heavy metals in investigations of other countries.

	V	Mn	Co	Ni	Cu	Zn	As	Cd	Pb	Reference
Surrounding area in this study	20.32	634.84	14.38	20.02	264.96	196.30	28.44	2.06	74.62	-
Bulgarian	n/a	n/a	8.00	17.00	1449.77	202.00	100.00	1.20	78.67	[12]
Legnica (Southwest Poland)	24	235	6.2	10.4	81	98	n/a	0.25	79	[13]

n/a, data not available

Table S8 The exposure level of the adults and children in the surrounding area and non-ferrous metal smelting plant (NMSP) for non- cancer risks assessment.

Element	$ADD_{s\text{-}oral}$		$ADD_{s\text{-}inh}$		$ADD_{s\text{-}dermal}$		ADD_s	
	adult	child	adult	child	adult	child	adult	child
Surrounding area								
Co	2.91E-06	3.31E-06	6.75E-10	7.55E-09	1.79E-10	1.40E-10	2.91E-06	3.32E-06
Ni	3.70E-06	4.21E-06	8.59E-10	9.60E-09	2.27E-10	1.78E-10	3.70E-06	4.22E-06
Cu	8.56E-05	9.75E-05	1.99E-08	2.22E-07	5.26E-09	4.13E-09	8.57E-05	9.77E-05
Zn	8.91E-05	1.01E-04	5.47E-09	4.29E-09	2.07E-08	2.31E-07	8.91E-05	1.02E-04
As	1.36E-05	1.54E-05	3.15E-09	3.52E-08	8.33E-10	6.53E-10	1.36E-05	1.55E-05
Cd	2.84E-06	3.24E-06	6.61E-10	7.39E-09	1.75E-10	1.37E-10	2.85E-06	3.25E-06
Pb	4.46E-05	5.07E-05	1.04E-08	1.16E-07	2.15E-09	2.74E-09	4.46E-05	5.08E-05
NMSP								
Co	8.86E-06	1.01E-05	2.06E-09	2.30E-08	5.45E-10	4.27E-10	8.86E-06	1.01E-05
Ni	5.04E-05	5.74E-05	1.17E-08	1.31E-07	3.10E-09	2.43E-09	5.05E-05	5.76E-05
Cu	4.64E-03	5.29E-03	1.08E-06	1.21E-05	2.85E-07	2.24E-07	4.64E-03	5.30E-03
Zn	7.03E-04	8.00E-04	4.32E-08	3.39E-08	1.63E-07	1.83E-06	7.03E-04	8.02E-04
As	4.06E-04	4.62E-04	9.43E-08	1.05E-06	2.49E-08	1.96E-08	4.06E-04	4.63E-04
Cd	4.64E-05	5.29E-05	1.08E-08	1.21E-07	2.85E-09	2.24E-09	4.65E-05	5.30E-05
Pb	4.44E-04	5.06E-04	1.03E-07	1.15E-06	2.14E-08	2.73E-08	4.45E-04	5.07E-04

Table S9 The exposure level of the adults and children in the surrounding area and non-ferrous metal smelting plant (NMSP) for cancer risks assessment.

Element	$ADD_{s\text{-}oral}$		$ADD_{s\text{-}inh}$		$ADD_{s\text{-}dermal}$		ADD_{s}	
	adult	child	adult	child	adult	child	adult	child
Surrounding area								
As	4.52E-06	1.29E-06	1.05E-09	1.93E-10	2.78E-10	5.44E-11	4.52E-06	1.29E-06
Cd	9.48E-07	2.70E-07	2.20E-10	4.05E-11	5.83E-11	1.14E-11	9.49E-07	2.70E-07
Pb	1.49E-05	4.23E-06	9.13E-10	1.79E-10	3.45E-09	6.34E-10	1.49E-05	4.23E-06
NMSP								
As	1.35E-04	3.85E-05	3.14E-08	5.77E-09	8.31E-09	1.63E-09	1.35E-04	3.85E-05
Cd	1.55E-05	4.41E-06	3.60E-09	6.61E-10	9.51E-10	1.86E-10	1.55E-05	4.41E-06
Pb	1.48E-04	4.22E-05	9.10E-09	1.78E-09	3.44E-08	6.32E-09	1.48E-04	4.22E-05

References

1. Wang, H. Z.; Cai, L. M.; Wang, Q. S.; Hu, G. C.; Chen, L. G., A comprehensive exploration of risk assessment and source quantification of potentially toxic elements in road dust: A case study from a large Cu smelter in central China. *Catena* **2021,** 196.
2. Müller, G., Index of geoaccumulation in sediments of the Rhine River. *Geol. J., 2* **1969,** pp. 108-118.
3. Sutherland, R. A., Bed sediment-associated trace metals in an urban stream, Oahu, Hawaii. *Environ Geol* **2000,** 39, (6), 611-627.
4. MEPC, *Technical guidelines risk assessment contaminated sites. China Environmental Press, Beijing* **2014**.
5. Duan, X. *Highlight of Chinese Children's Exposure Factors Handbook;* China Environmental Press: Beijing, China, 2016.
6. Duan, X. *Summary of Chinese Population Exposure Parameters Manual*; China Environmental Press: Beijing, China, 2014.
7. Jiang, Y.; Chao, S.; Liu, J.; Yang, Y.; Chen, Y.; Zhang, A.; Cao, H., Source apportionment and health risk assessment of heavy metals in soil for a township in Jiangsu Province, China. *Chemosphere* **2017,** 168, 1658-1668.
8. USEPA, *Environmental Protection Agency, Region 3, Risk Based Screening Tables (RSLs).* Unites States Environmental Protection Agency, Washington, DC. **2021**.
9. Ferreira-Baptista, L.; De Miguel, E., Geochemistry and risk assessment of street dust in Luanda, Angola: A tropical urban environment. *Atmospheric Environment* **2005,** 39, (25), 4501-4512.
10. MEEPRC. *Soil Environmental Quality-Risk Control Standard for Soil Contamination of Agricultural Land (GB15618-2018)*; Ministry of Ecology and Environment of the People's Republic of China: Beijing, China, 2018.
11. MEEPRC. *Soil Environmental Quality-Risk Control Standard for Soil Contamination of Development Land (GB36600-2018)*; Ministry of Ecology and Environment of the People's Republic of China: Beijing, China, 2018..
12. Jordanova, N.; Jordanova, D.; Tcherkezova, E.; Georgieva, B.; Ishlyamski, D., Advanced mineral magnetic and geochemical investigations of road dusts for assessment of pollution in urban areas near the largest copper smelter in SE Europe. *Sci Total Environ* **2021,** 792, 148402.
13. Samecka-Cymerman, A.; Stankiewicz, A.; Kolon, K.; Kempers, A. J., Bioindication of Trace Metals in Brachythecium rutabulum Around a Copper Smelter in Legnica (Southwest Poland): Use of a New Form of Data Presentation in the Form of a Self-Organizing Feature Map. *Arch Environ Con Tox* **2009,** 56, (4), 717-722.
